# Burden and Historical Trend of Buruli Ulcer Prevalence in Selected Communities along the Offin River of Ghana

**DOI:** 10.1371/journal.pntd.0004603

**Published:** 2016-04-14

**Authors:** Kobina Assan Ampah, Prince Asare, Daniel De-Graft Binnah, Samuel Maccaulley, William Opare, Katharina Röltgen, Gerd Pluschke, Dorothy Yeboah-Manu

**Affiliations:** 1 Noguchi Memorial Institute for Medical Research, University of Ghana, Legon, Ghana; 2 Swiss Tropical and Public Health Institute, Molecular Immunology, Basel, Switzerland; 3 University of Basel, Basel, Switzerland; 4 Department of Earth Science, University of Ghana, Legon, Ghana; 5 National Buruli Ulcer Control Programme, Disease Control Unit- GHS, Accra, Ghana; University of Tennessee, UNITED STATES

## Abstract

Buruli ulcer (BU) is a neglected tropical skin disease caused by *Mycobacterium ulcerans* with more than two thirds of the global cases reported in West Africa. A nationwide active BU case search conducted in 1999 identified two health districts along the Offin River as two of the three most endemic districts in Ghana. Based on recent anecdotal accounts that transmission is unstable along the Offin River, we conducted from March to June 2013 an exhaustive household survey and active case search in 13 selected communities within a five-kilometer radius along the Offin River. The overall prevalence of BU was 2.3% among the surveyed population of 20,390 inhabitants and 477 of the total 480 cases detected (99.4%) were historical (healed) cases. By estimating the year of occurrence for each case per community and taking into account available passive surveillance records of health facilities and the District Health Directorate, we observed a general trend of continuous emergence of cases in communities located midstream the Offin River whereas downstream communities showed more sporadic patterns. We monitored the incidence of cases after the survey and recorded a cumulative incidence rate of 0.04% for the 13 communities over a 17-month active surveillance period from August 2013 to December 2014. Our data reveal an overall decline in BU incidence along the Offin River similar to the general decline in BU incidence in recent years reported by the World Health Organization for West Africa.

## Author Summary

Buruli ulcer (BU) is a tropical skin disease caused by *Mycobacterium ulcerans* and more than two thirds of the global cases reported in West Africa. The Offin has been considered the most endemic river valley in Ghana following a nationwide active case search conducted in 1999. Here, we present findings of an exhaustive household survey and case search of 13 selected communities along the Offin river aimed at addressing recent anecdotal accounts of unstable transmission of *M*. *ulcerans* within the river basin. We observed among the surveyed population of 20,390 inhabitants, an overall 2.3% prevalence of BU with 99.4% of the total cases detected being historical cases. We also observed a general trend of continuous and sporadic emergence of cases in mid and downstream communities, respectively. Subsequently, we detected a total of eight cases (0.04% cumulative incidence rate) in a prospective 17-month active surveillance of all 13 communities. These data confirm the recent decline in BU incidence in historically endemic communities along the Offin river basin, analogous to the observation made in recent years by the World Health Organization for West Africa.

## Introduction

Buruli ulcer (BU) is a necrotizing skin disease caused by *Mycobacterium ulcerans* [1]. The disease has been reported in over 30 countries worldwide, mainly in the tropics, but the brunt of it seems to be mainly experienced in West Africa with Côte d’Ivoire, Ghana, Benin and Cameroon reporting more than 80% of the global number of cases [2]. Within the endemic countries, BU occurs in foci typically affecting inhabitants of impoverished and rural settings where access to medical care is a big challenge [3].

Control of BU is based mainly on early case detection and adequate antibiotic treatment and wound management. The current treatment regimen recommended by the World Health Organization (WHO) includes daily administration of streptomycin and rifampicin for eight weeks [4]. Advanced lesions may require debridement and/or skin grafting as an adjunct to improve healing and to prevent or correct deformities [5–7]. Nevertheless BU treatment is often associated with long hospital stays and represents a major socio-economic burden in the affected communities [8].

**Fig 1:**
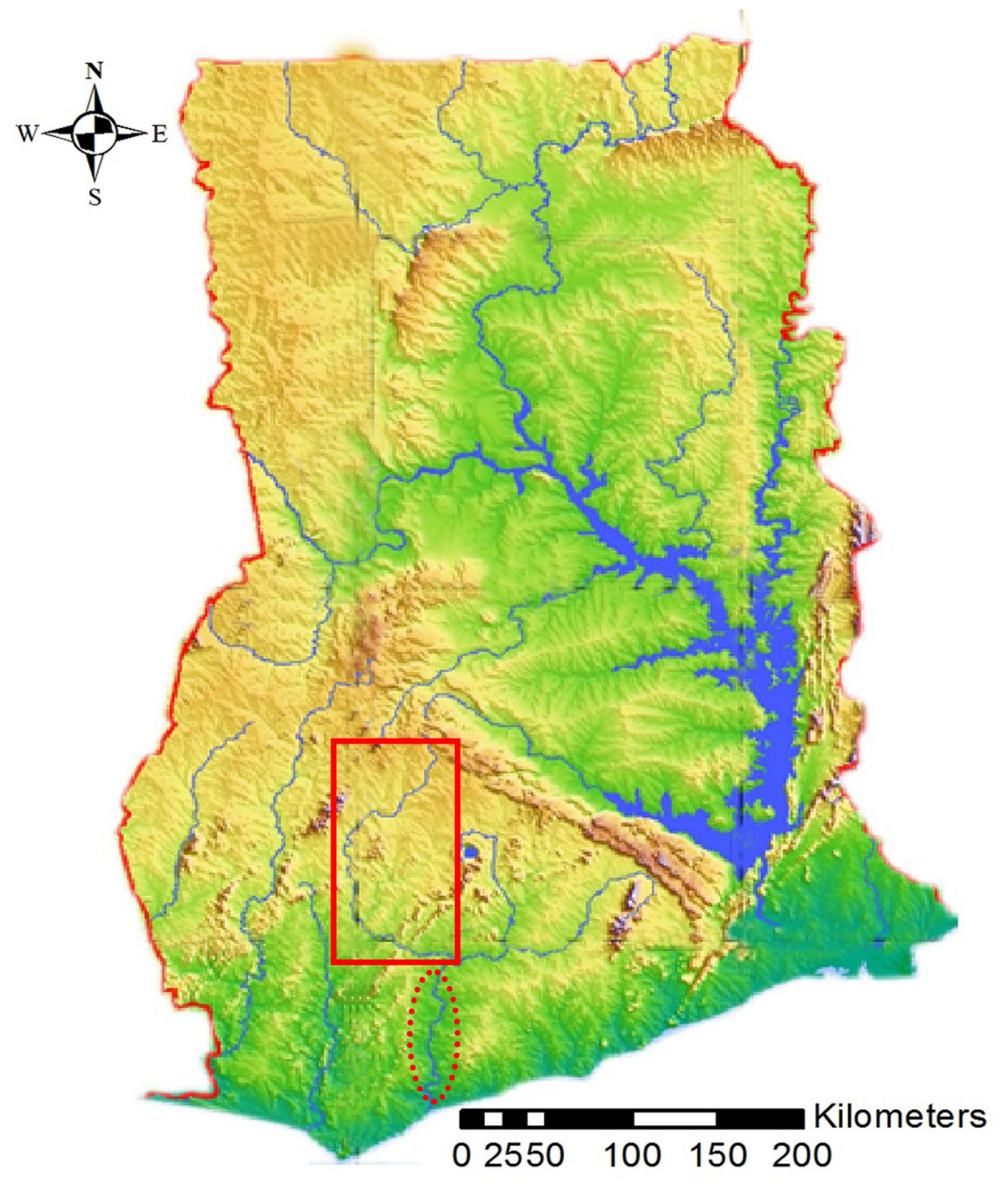
Study area. Map of Ghana showing major river systems. Our study area comprised part of the Offin river basin, shown here with a red rectangle. The Pra River is indicated by the dotted line.

In Ghana, nearly 1,200 BU cases were reported between 1993 and 1998 by the first passive surveillance system established in the country and between 2004 and 2014 more than 9,000 cases have been reported. A nation-wide active case search conducted in 1999 resulted in an overall crude prevalence rate (clinically diagnosed active lesions) of 20.7 per 100,000 inhabitants [9]. The most endemic district identified within this study was the Amansie West District, which is drained by the Offin river. The river takes its source from the Mampong scarp in the Ashanti region, flows from Boanim community through Bipoa community and eventually joins the Pra river in the Central Region (Fig 1). With farming and alluvial gold mining being the characteristic human activities in the river basin, spatio-epidemiological studies have consistently associated these activities with BU [10,11].

From June to July 2012, we paid reconnaissance visits to all 11 Health Districts drained by the Offin River. Anecdotal accounts gathered from interactions with local health staff and leaders of some selected communities indicated the absence of recent (in the past 3 to 5 years) cases in some communities historically known to be BU endemic and the emergence of new cases in some non-endemic communities. Since BU case data at the local and district health facilities were scanty, we set up this study with the following specific objectives: i) to determine the prevalence of BU in 13 selected communities in the Offin river basin, ii) to characterize the retrospective occurrence of BU cases for each community based on active surveillance data and available case data at the district and local health centers. We also report on active surveillance activities conducted to prospectively monitor the emergence of new BU cases in the selected communities.

## Materials and Methods

### Ethics statement

Ethical clearance for this study was obtained from the institutional review board of the Noguchi Memorial Institute for Medical Research, NMIMR, with a federal wide assurance number FWA00001824 and the ethics review committee of the Ghana Health Service (ethical approval ID number GHS-ERC:06/07/13). Participation in all aspects of the study was voluntary and all confirmed cases—independent of their participation- were treated according to the treatment guidelines established by the National Buruli Ulcer Control Program (NBUCP). Written informed consent was obtained from all patients before their lesions were sampled for laboratory diagnosis. Parents or guardians provided written consent on behalf of all child participants.

### Study area

This study was conducted in the Offin river valley of Ghana (Fig 1). The Akans, who form the largest ethnic group in Ghana, are the main inhabitants of five out of the ten administrative regions (Ashanti, Central, Western, Eastern and Brong–Ahafo). The Offin river drains two of these regions namely the Ashanti and Central Regions. Due to the intense gold mining activities carried out in the river basin, anecdotal accounts suggest that this preponderance breaks down at the community level due to the influx of migrants from other parts of Ghana and neighboring West African countries.

**Fig 2:**
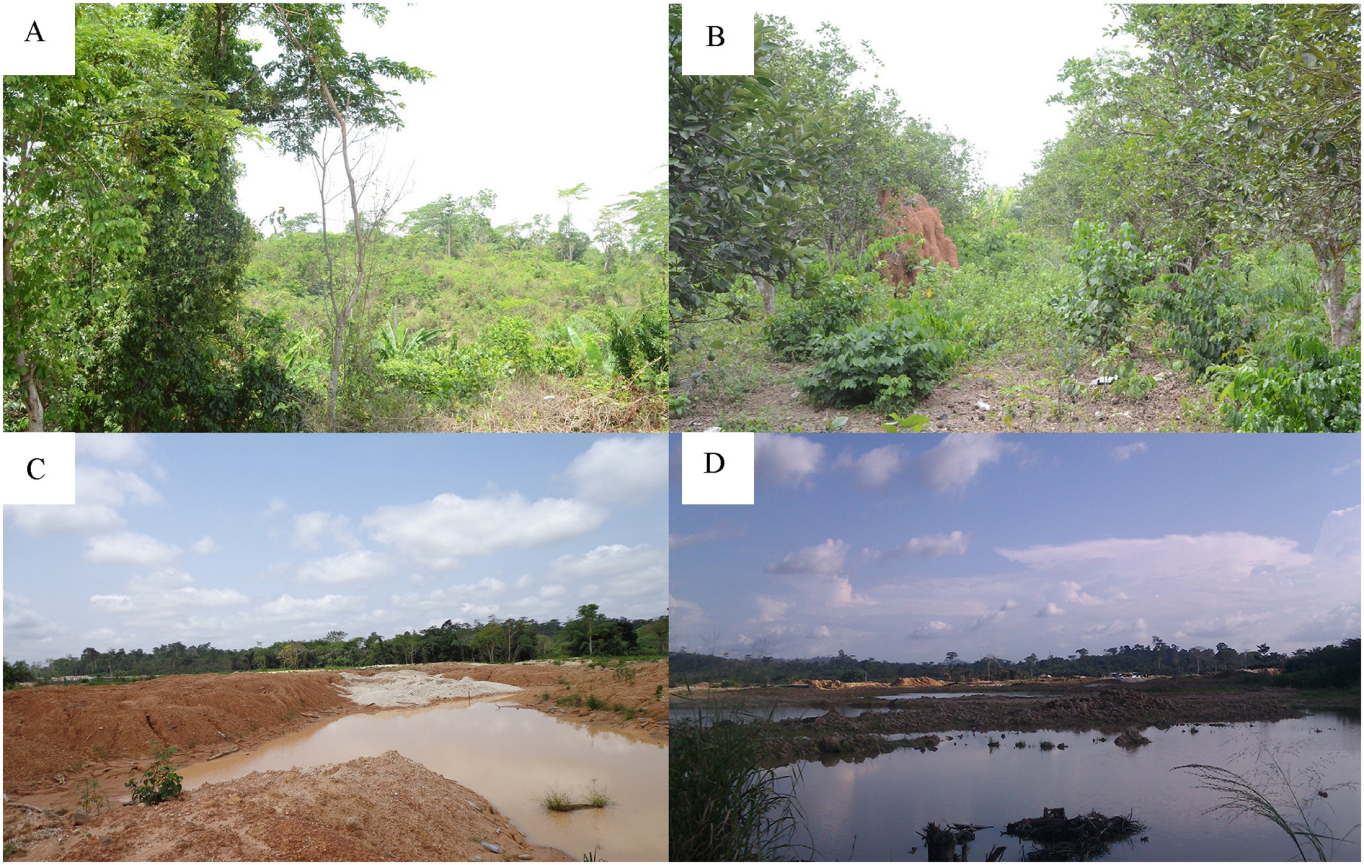
Land cover and use in selected communities along the Offin river. Upstream communities (**A**) Bedomase and **(B**) Krakrom, mid-stream community (**C**) Ntobroso and downstream community (**D**) Pokukrom.

**Fig 3:**
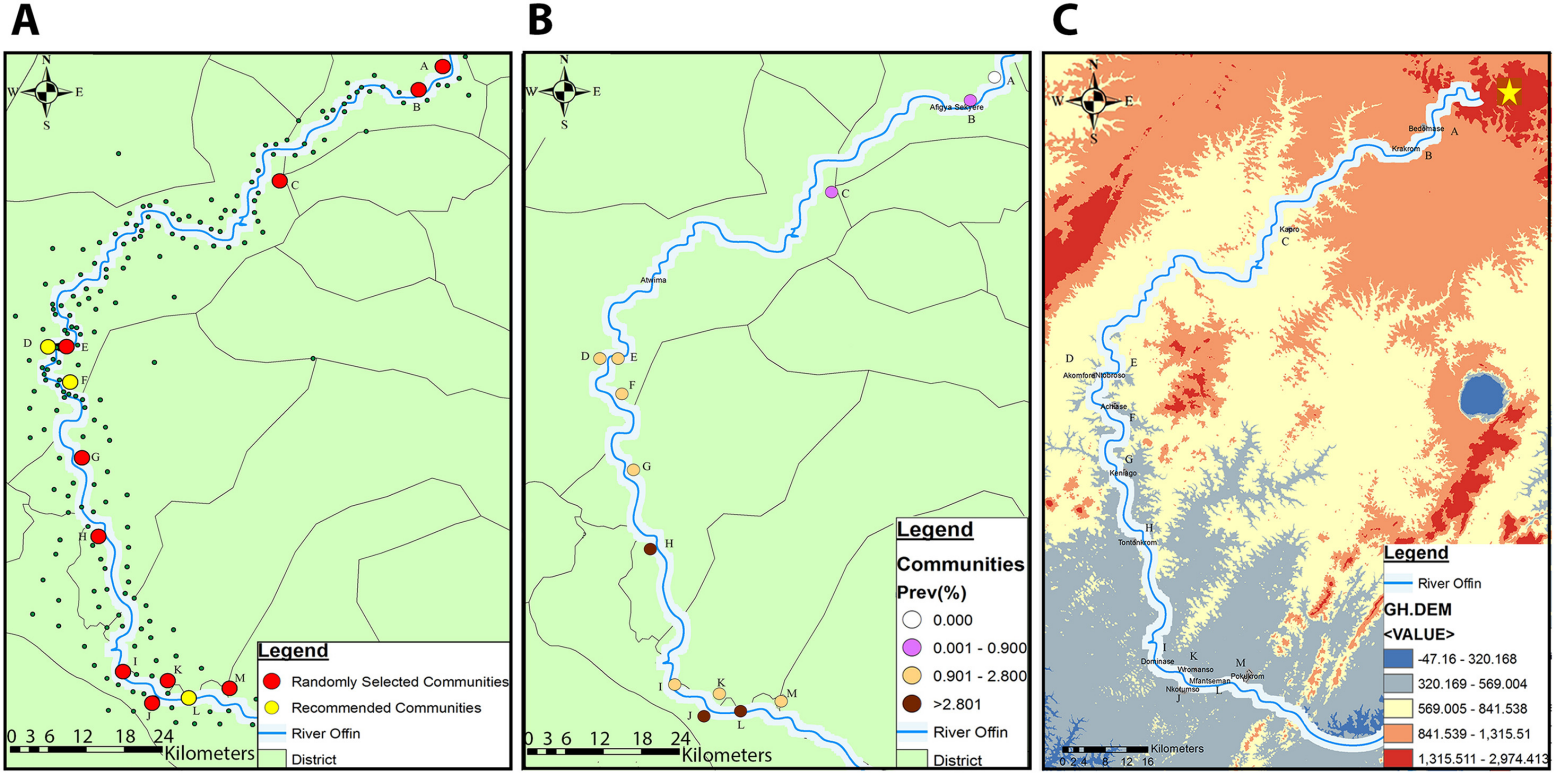
Elevation and distribution of Buruli ulcer burden along the Offin River. (A) A map showing communities located within the 5 km buffer along the Offin river. The 10 red colored study communities were randomly selected, the three yellow colored communities were added to the study based on recommendations by local health staff. (B) Spatial distribution of Buruli ulcer prevalence along the Offin river. (C) Spatial altitudes variation (indicated in feet) along the Offin river. The Mampong highland area is indicated by the yellow star. Community codes; Bedomase (A), Krakrom (B), Kapro (C), Akomfore (D), Ntobroso (E), Achiase (F), Keniago (G), Tontonkrom (H), Dominase (I), Nkotumso (J) Wromanso (K), Mfantseman (L) and Pokukrom (M).

Our study area presents major landscape differences between communities located upstream the river on one hand, and those located mid and downstream on the other hand. As illustrated in Fig 2, land cover of upstream communities Bedomase (A) and Krakrom (B) was mainly farmland. In contrast, the peripheries of the mid and downstream communities were generally characterized by heavy mining activities as exemplified by Ntobroso (C) and the downstream community Pokukrom (D). In addition, our assessment of the elevation patterns with respect to the Offin river course revealed that the highlands of Mampong in the Ashanti region from which the river takes its source were at least 401m (1,315 ft) high (Fig 3C). Thus communities A to C located upstream the river were situated in areas with altitudes of at least 256m. Conversely, the midstream to downstream region of the Offin river recorded relatively lower elevation values ranging from 173m to 98m (569–320 ft).

Following a nation-wide active BU case search [9], the NBUCP maintained a database of geographic co-ordinates of communities visited. We obtained from the NBUCP geographic co-ordinates of all communities in the 11 health districts of the Offin river valley. Using the ArcGIS 10.0 mapping software, we created a buffer of five kilometers along the river. This seeded a total of 199 communities from which we selected 10 by simple randomization using a randomization tool embedded in the software (Fig 3A). The selected communities were Bedomase (A) and Krakrom (B) of the Sekyere south district, Kapro (C) of the Atwima Nwabiagya district, Ntobroso (E) of the Atwima-Mponua district, Keniago (G) and Tontonkrom (H) of the Amansie West district, Dominase (I) and Nkotumso (J) of the Upper Denkyira West district, Wromanso (K) of the Amansie Central district and Pokukrom (M) of the Upper Denkyira East district (Fig 3A). Based on recommendations of local health staff, three additional communities known to be BU endemic were included to bring the total number to 13. These communities were; Akomfore (D) and Achiase (F) of the Atwima Mponua district, and Mfantseman (L) of the Upper Denkyira East district. Other selected communities known to be endemic based on available passive surveillance records were Ntobroso (E), Keniago (G), Tontonkrom (H), Dominase (I), Wromanso (K), Pokukrom (M) and Nkotumso (J). Passively, no BU case has ever been reported for Bedomase (A) and Krakrom (B). We were unable to substantiate the endemicity of Kapro (C) prior to the survey.

### Community entry

Entry into each community was carried out by a team consisting of a research assistant from the NMIMR, a field officer from NBUCP, a local disease control officer (DCO) and a community based surveillance volunteer (CBSV). We met chiefs, traditional and opinion leaders of each community to whom we explained the structure, aims and benefits of the study. Once we received their approval, information on the study was relayed to the community members through the CBSV and community information delivery systems.

### Data collection

Between February and May 2013, we conducted an exhaustive household survey and active case search for BU. We formed teams each consisting of one research assistant from NMIMR, one local health staff routinely involved in the management of BU cases and one community volunteer. To aid in the description of the disease to participants being interviewed, each team was equipped with posters and picture charts illustrating the clinical forms of BU. Members of the teams received training on the clinical signs of BU, how to fill the survey forms and also how to take GPS co-ordinates. The teams went out to each inhabitable structure, numbered them serially and interviewed all inhabitants who were present. Households of inhabitants who were absent were noted and in two follow-up sessions on study participants (July and December 2013), we interviewed those absentees who were now available. Information on minors was provided by their parents or guardians. In addition to demographic data, clinical data were also collected for detected cases and GPS co-ordinates for every household.

### Definition of variables

An inhabitant was classified as a part-time resident of a community if in the past year prior to the survey the person; a) travelled and stayed out of the community for more than 3 months and b) had his livelihood (such as occupation and education) in another community such that he spent more than 6 hours of his day-time there. A full-time resident on the other hand a) never travelled out of the community in the past year or b) travelled but stayed no longer than 3 months and c) had his livelihood in the community being surveyed and thus spent more than 6 hours of his day time there.

Each time we completed surveying a community, we extracted information on inhabitants with suspected lesions and revisited them together with a clinician of extensive experience in diagnosing BU. The clinician then examined the lesions and diagnosed them as either active or inactive (healed) BU or as BU-unrelated lesions. Generally, depressed stellate scars were anecdotally considered as healed BU lesions whereas healed lesions with cleared skin areas were considered as BU-unrelated. Thus, cases with both active and healed lesions were included in this study. Clinically diagnosed active lesions were microbiologically confirmed by the detection of *M*. *ulcerans* insertion sequence (IS) *2404* by diagnostic PCR. In addition, BU lesions were clinically classified according to the WHO guidelines. Category I lesion was defined as a single lesion of size less than or equal to five centimeters at the widest diameter, category II as a single lesion between five and 15 cm and category III as either a single lesion greater than 15 cm or multiple lesions or a lesion at critical areas of the body.

### Estimation of the year of emergence of BU cases in each community

Using posters and charts the clinical features of BU was described to the participants after which, we asked participants if they have ever had an episode of BU at any point in their lives. This was done to determine the overall crude life time prevalence of BU in the population surveyed. However, in order to assess the historical emergence of cases for each community, we restricted our analysis of the healed cases to a 24 year prevalence period (1990 to 2013) and compared our data with passive surveillance records obtained from 2000 to 2013.

Admittedly, such retrospective analyses are prone to recall bias particularly for BU where the appearance of symptoms does not reflect the moment of contracting the disease due to a relatively unknown incubation period. For BU cases with healed lesions, we estimated the year of developing the symptoms of the disease by i) examining health records and BU 01 forms (if available) and ii) verbally interviewing the cases and confirming from at least two other household inhabitants or nearby neighbors who were present and witnessed the case having the disease. To facilitate recall in healed cases of more than one year history, estimation was done by reference to any other household member or close neighbor who was born around the period the case developed symptoms of the disease. Once this estimation was confirmed by two other inhabitants present at the time of developing the clinical symptoms of BU, the period was estimated and noted using the year of birth of the inhabitant referenced.

### Active surveillance of BU

Subsequent to the exhaustive household survey, we continued to monitor the emergence of BU cases using two approaches: i) community outreach program and ii) monthly household visits by community volunteers. We conducted the community outreach once every three months in all 13 selected communities. Specifically in the months of July 2013, September 2013, December 2013 to January 2014, March 2014, July to August 2014 and November 2014. This program involved one evening of educating the community members on transmission, early case detection and treatment by showing BU documentaries and interacting with them through questions and answers. The following morning, the inhabitants were screened and those with clinically suspected BU lesions were sampled for laboratory confirmation.

For seven of the 13 communities (Achiase (F), Ntobroso (E), Akomfore (D), Keniago (G), Pokukrom (M), Wromanso (K) and Mfansteman (L)), we employed a monthly household-visit based surveillance as an additional tool to the surveillance by the community outreach program. All seven communities were selected based on the willingness of the CBSV to voluntarily carry out the exercise. We trained and equipped one CBSV from each of the afore-mentioned communities with android phones (HTC wildfire S) pre-loaded with a BU surveillance questionnaire. The questionnaire was designed using the Epicollectplus mobile application (http://plus.epicollect.net/). From August 2013 to December 2014, the CBSVs were mandated to visit all households in a month and record any suspected case using the mobile application and a notebook. Suspected cases were then sampled by a local health staff and the samples were sent to the NMIMR for laboratory confirmation. We then assessed the feasibility and efficiency of this approach by evaluating the number of cases detected, the severity of lesions sampled and the community coverage (the proportion of households inventoried during the exhaustive survey that could be visited in a month by the volunteer).

### Laboratory confirmation of BU cases

To confirm suspected active BU lesions, two swab specimens were collected from the undermined edges of ulcerative lesions. For pre-ulcerative lesions, one fine needle aspirate (FNA) was transferred into 500 μl phosphate buffered saline (PBS) as previously described [12] and transported to NMIMR at 4°C. Laboratory confirmation was conducted as previously described. Briefly, suspensions from pooled swabs of the same lesion containing 2 ml of PBS were concentrated by centrifuging at 3,000 x g for 15 minutes. The supernatant was then decanted and the sediment mixed with PBS to make up a final volume of 500 μl suspension from which 100 μl was used to prepare slides for Ziehl-Neelsen (ZN) microscopy. From the residual 400 μl suspension, we extracted *M*. *ulcerans* DNA for IS*2404* PCR.

### Mapping and data analysis

Summary data from household surveys were mapped and analyzed using the ArcMap (Economic and Social Research Institute, version 10.0). The elevation values (presented in feet) were derived from 15 meter resolution Advanced Spaceborne Thermal Emission and Reflection (ASTER) Satellite digital elevation model (DEM) data, obtained at a sun angle of 59.6 degrees.

All statistical analyses were carried out using GraphPad Prism version 6.0 (GraphPad Software, San Diego California USA) and Stata 12 (Statacorp 2011 statistical software Release 12. College Station, TX: StataCorp LP).

## Results

### Demographic characteristics of the surveyed population

A total of 2,822 households of three hamlets, seven villages and three peri-urban settings along the Offin river were visited to survey the population for BU.

**Fig 4:**
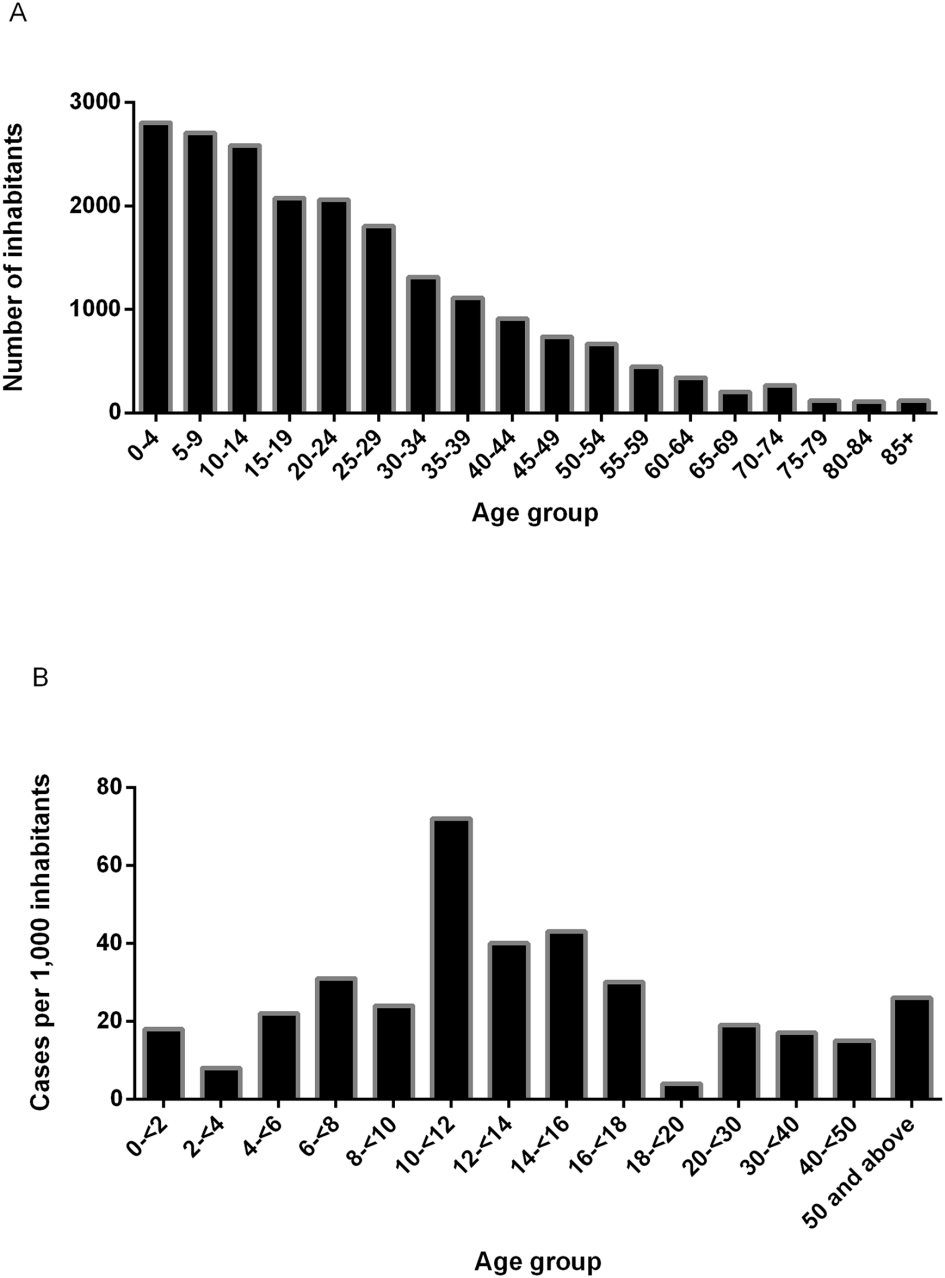
Population age distribution and age adjusted prevalence of BU in the surveyed population. **(A)** Age distribution of the surveyed population (n = 20,390 inhabitants) of the 13 selected study communities in the Offin river basin. **(B)** Age distribution of BU cases based on the age of the 480 identified cases at onset of the disease adjusted for age (per 1,000 inhabitants) using the age structure of the general surveyed population.

In all, we surveyed a total population of 20,390 inhabitants, 50.08% (n = 10,211) females and 49.92% (n = 10,179) males (Table 1). The majority (90.0%) of these inhabitants were full time residents. Characteristic of a youthful population, the mean age recorded for the survey was 23.6 (S.D +/- 18.8) years and 39.7% of the surveyed population below the age of 15 years (Fig 4A). As expected, in all of the visited communities, the Akans represented the major ethnic group (46.9% to 96.5%). 82.8% of the population between the ages of 4 and 18 years were students. We observed that miners formed less than 1% of the population we surveyed upstream the river (0.4% from Bedomase and none from Krakrom and Kapro). On the other hand, miners were well represented in mid and downstream communities like Nkotumso and Tontonkrom where they formed the highest and second highest occupational group, respectively.

**Table 1:** Demographic characteristics of the surveyed population.

	Community													
Demogr aphic variables	A	B	C	D	E	F	G	H	I	J	K	L	M	Total
**Population (n)**	1,688	111	692	1,016	1,949	1,900	3,350	2,945	2,802	2,518	216	303	900	20,390
**Type of settlement**	Peri-urban	Hamlet	Village	Village	Village	Village	Village	Village	Peri-urban	Peri-urban	Hamlet	Hamlet	Village	
**Households**														
**Inhabitants per household**	1–25	2–17	2–25	1–20	1–30	1–21	1–32	1–25	1–27	1–30	1–20	1–10	1–19	1–32
**Total number of households**	226	16	64	157	251	295	413	399	384	370	41	85	121	2,822
**Residents**														
**Full time (%)**	87.5	84.7	93.8	89.1	84.8	92.2	86.3	93.1	92.1	93.2	74.1	90.8	91.3	90
**Part time (%)**	12.5	15.3	6.2	10.9	15.2	7.8	13.7	6.9	7.9	6.8	25.9	9.2	8.7	10
**Total**	100	100	100	100	100	100	100	100	100	100	100	100	100	
**Sex**														
**Females, n (%)**	876 (51.9)	65 (58.6)	363 (52.5)	499 (49.1)	972 (49.9)	939 (49.4)	1,719 (51.3)	1,467 (49.8)	1,423 (50.8)	1,232 (48.9)	106 (49.1)	141 (46.5)	409 (45.4)	10,211
**Age range**	0.2–110	0.5–72	0.2–100	0–120	0.2–115	0.1–120	0–120	0.1–114	0–110	0–100	1–85	0.3–90	0.1–99	0–120
**Mean age in years (S.D)**	24.3 (20.7)	22.4 (17.4)	24.0 (19.9)	23.1 (18.8)	24.4 (18.9)	25.9 (20.7)	22.9 (19.4)	22.2 (16.6)	22.9 (17.9)	23.3 (17.7)	27.0 (20.3)	23.3 (18.0)	24.3 (18.4)	23.6 (18.8)
**Ethnicity (%)**														
**Akan**	88	53.5	96.5	79	94.2	90.4	46.9	71.6	82.6	68.2	53.8	73.6	92.1	75.8
**Ewe**	0.3	3.3	0	8.7	0.1	1.2	0.9	6.6	9.6	14.5	30	2.1	6	5.4
**Mole**	0.3	0	0.1	0.1	0	0.1	0	0	0.2	0.6	0	0.3	0	0.1
**Ga / Ada**	0.2	1.2	0	3	0.4	0.2	0	0.7	1.4	1.6	1	0.5	0.5	0.8
**Guan**	0.1	0.1	0	0	0	0	0	0	0.1	0	0	0.3	0	0
**Gruma**	1.6	5.4	0	0	0	0.3	1.8	1	0.1	0.5	2	0.4	0	0.7
**Grusi**	0	0.1	0.1	0.2	0	0.4	0	0	0.7	0.9	0	2.6	0	0.3
**Mande**	0.1	0.1	0	0	0.1	0	0	0	0	0	0.1	0.5	0	0
**Mamprusi**	0	0.2	0	0.1	0.3	0.2	0	0	0	0.1	0.1	0.8	0	0.1
**Kussasi**	3.9	13.3	2.4	1.8	0.4	3.9	31.5	5.3	2.7	2.8	4.2	3.7	0	7.7
**Others**	5.5	22.8	0.9	7.1	4.5	3.3	18.9	14.8	2.6	10.8	8.8	15.2	1.4	9
**Total**	100	100	100	100	100	100	100	100	100	100	100	100	100	
**Main Occupation (%)**														
**Unemployed (children and elderly)**	12.6	15.3	16.5	17.1	16.7	13.8	20.9	15.1	12.8	13.4	12.1	13.2	13.8	14
**Unemployed (Adult)**	6.8	1.8	6.4	4.2	4.4	3.2	6.2	3	14.2	8.6	2.8	4	8.2	8
**Student**	42.4	38.8	36.7	32	28.7	33.1	33.8	28.5	22.7	12.8	29.6	32	28.4	28
**Fisherman**	0	0	0	0.1	0.2	0	0.1	0	5.2	18.4	0.5	0	0	0
**Farmer**	21	38.7	24.4	36.1	28.3	31.3	17.5	18	14	9	36.1	40.9	21	21
**Miner**	0.4	0	0	4.7	8.8	9.5	12.2	24.4	14.7	18.9	9.7	5.6	17.1	17
**Skilled laborer**	7.7	1.8	3	2	5.2	3.8	3.2	2.9	5.8	4	3.2	1.3	3	3
**Unskilled laborer**	8.8	3.6	11.7	2.5	6.3	4.6	5	7	9.4	13.5	4.6	2.3	7.2	7
**Others**	0.3	0	1.3	1.3	1.4	0.7	1.1	1.1	1.2	1.4	1.4	0.7	1.3	1
**Total**	100	100	100	100	100	100	100	100	100	100	100	100	100	
**BU Prevalence(%)**														
**Healed cases**	0	0.9	0.1	3	1.6	3.8	1.2	3.2	2.5	3.6	2.3	8.9	1.3	2.3
**Active cases**	0	0	0	0.1	0.1	0.1	0	0	0	0	0	0	0	0

Community codes: Bedomase **(A)**, Krakrom **(B)**, Kapro **(C)**, Akomfore **(D)**, Notbroso **(E)**, Achiase **(F)**, Keniago **(G)**, Tontonkrom **(H)**, Dominase **(I)**, Nkotumso **(J)** Wromanso **(K)**, Mfantseman **(L)** Pokukrom **(M)**

### BU cases identified in the selected communities

Based on clinical signs and symptoms, we identified seven suspected active BU cases of which three were confirmed; one by both ZN and PCR and two by PCR only. All three confirmed cases (one male and two females) had ulcerative lesions. The male patient who was 6 years old presented with a category I ulcer on his thorax. One 35 year old female presented with category I on the upper left limb and the other 22 year old female had a category II ulcer on her lower left limb. Based on the clinical assessment of healed lesions by an experienced clinician, we could detect a total of 477 cases with healed BU lesions from the 13 communities. Of these 477 healed cases, 138 (28.9%) had been previously diagnosed (clinical and/or laboratory) as active BU and noted in existing passive surveillance records. We estimated 0.01% and 2.34% prevalence of active and healed BU cases respectively in the surveyed population. We observed no significant difference (P = 0.12) between the proportion of historical cases in females (2.6%, 266/10209) when compared to the proportion recorded for males (2.0%, 211/10178). The age of the 477 healed cases identified during the survey at the estimated onset of the disease ranged from 0.75 to 87 years with a mean age of 31 years (SD = 18). Children under 15 years accounted for 48.1% of the total number of cases. When we computed the age adjusted prevalence based on the age distribution of the population surveyed, we observed the highest peak for children between 10 and 12 years (Fig 4B).

### Emergence of BU cases in the community and distribution of burden along the Offin river

The BU prevalence for all surveyed communities is listed in Table 1. The disease burden was not uniform along the river. By grouping all 13 study communities according to their location along the Offin river, we observed that BU cases detected formed 0.1% (2 out of 2,491) of the total population surveyed upstream the river (communities A to C). This was significantly smaller (P<0.001) than the proportion recorded for communities D to H located mid-stream (2.4%, 274/11,160) and I to M located down-stream the river (3.0%, 204/6,739). As illustrated in Fig 3B, upstream, we detected no case in Bedomase (A) but recorded a prevalence of 0.90% and 0.14% in Krakrom (B) and Kapro (C), respectively. Prevalence was higher midstream (between 1.22% and 3.89%). The highest overall prevalence (8.9%) was recorded downstream the river in the Mfantseman (L) community. In the other downstream communities we recorded a prevalence of 2.46%, 3.61%, 2.31% and 1.33% for Dominase (I), Nkotumso (J), Wromanso (K) and Pokukrom (M), respectively.

**Fig 5:**
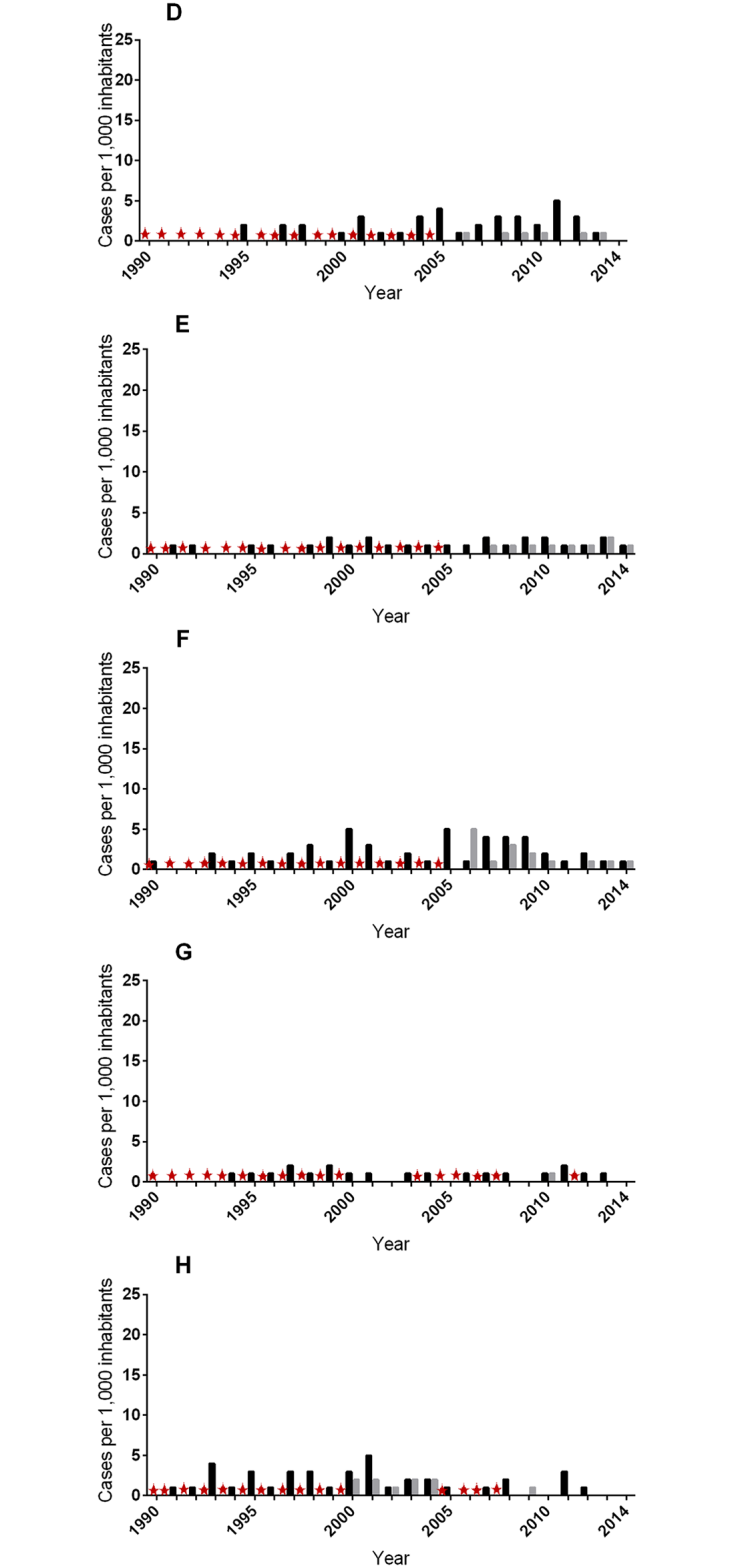
Estimated annual case trend for communities located mid-stream of the Offin river. Trends were reconstructed using data from active survey (ACS) and passive surveillance data (PCS) available at the local and district health facilities. The red star represents unavailable passive surveillance data for a particular year. Community codes: **D** (Akomfore), **E** (Ntobroso), **F**(Achiase), **G** (Keniago), **H** (Tontonkrom). No case was detected in Bedomase (**A**) and index cases were detected in Krakrom (**B**) and Kapro (**C**).

**Fig 6:**
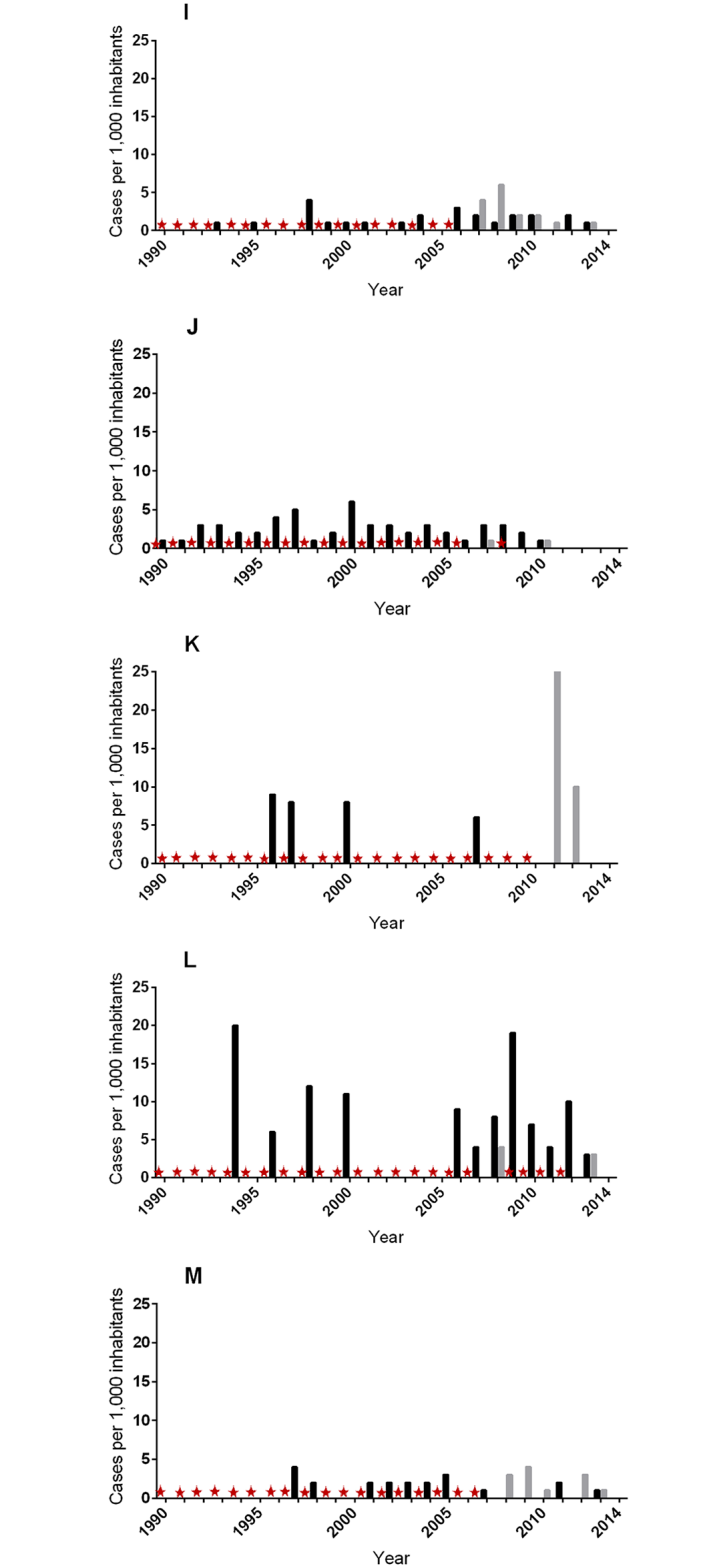
Estimated annual case trend for communities located down-stream of the Offin river. Trends were reconstructed using data from active survey (ACS) and passive surveillance data (PCS) available at the local and district health facilities. The red star represents unavailable passive surveillance data for a particular year. Community codes: **I** (Dominase), **J** (Nkotumso), **K** (Wromanso), **L** (Mfantseman), **M** (Pokukrom).

Based on the BU history data compiled for all 480 cases detected during the active case survey (ACS), we estimated the time span during which cases emerged for each community included in the survey. In a second step, we compared our results with the annual passive case surveillance (PCS) data available at the local and district health centers. We estimated a population growth rate of 3.8% per annum for communities A to H of the Ashanti region and 3.2% per annum for communities I to M of the Central region using data from the regional population census conducted in 2000 and 2010. The annual BU case estimates for both ACS and PCS were expressed per 1,000 inhabitants to facilitate the comparison of trends between communities (Figs 5 and 6).

While PCS data prior to the year 2000 was not available for any of the 13 communities surveyed, our annual ACS trend corroborated reasonably well with PCS data available for the years later than 2000. Altogether, we observed slightly differing trends in BU case emergence for upstream and downstream communities. The most upstream-localized community Bedomase (A) was non-endemic, as no case has ever been detected by PCS or ACS. In upstream communities Krakrom (B) and Kapro (C), we identified only one index case each corresponding to nine cases per 1,000 inhabitants and one case per 1,000 inhabitants respectively. While no PCS data was available for both cases detected, the cases were estimated to have emerged in 2009 and 2005 for Krakrom (B) and Kapro (C) respectively. Among all the mid-stream communities (D-H), we observed a continuous emergence of BU cases with at least one case per 1,000 inhabitants being recorded each year by either ACS or PCS or both from 2000 to 2014 (Fig 5). The downstream communities (I to M) which incidentally represent communities located within the Central region were generally characterized by a less continuous trend (Fig 6). For the historically endemic community Nkotumso (J), we observed an absence of cases from 2010 to 2014 after a long period of rather consistent emergence of cases. The further downstream communities Wromanso (K) and Mfansteman (L) were characterized by sporadic emergence of cases. Additionally, the highest number of cases per 1,000 inhabitants for a single year (25 cases in 2011) was observed in Wromanso (K).

In all, 95.8% (460/480) of the total BU cases detected have resided in their respective communities for more than three months. Additionally, when we stratified the population surveyed by their length of stay in the communities, we observed that BU cases made up 3.2% (460/14,332) of the group with more than three months of residence. This was significantly higher (P<0.001) when compared to the proportion recorded for those who have resided for three months or less (0.6%, 20/3613).

### Active BU surveillance

By employing both community outreach and household-visit surveillance, we continued to monitor the emergence of BU cases in all 13 selected communities over the 17-month period from August 2013 to December 2014. In all, we detected 29 clinically suspected BU cases during this active surveillance period. Five of these cases were detected through the community outreach program (four from Ntobroso and one from Dominase) and 24 were detected through the monthly household visits. Eight of them (27.6%) were laboratory confirmed by IS*2404* PCR. As expected for an active surveillance activity, all eight cases (five males and three females aged between 3 and 35 years, with a mean age of 18 (SD = 9))) had lesions in their early stages; five were detected with pre-ulcerative lesions (four nodules and one plaque) and three presented with Category II ulcers. The 21 non-confirmed suspected BU cases were referred to the district hospital for further assessment and alternative diagnosis.

**Fig 7:**
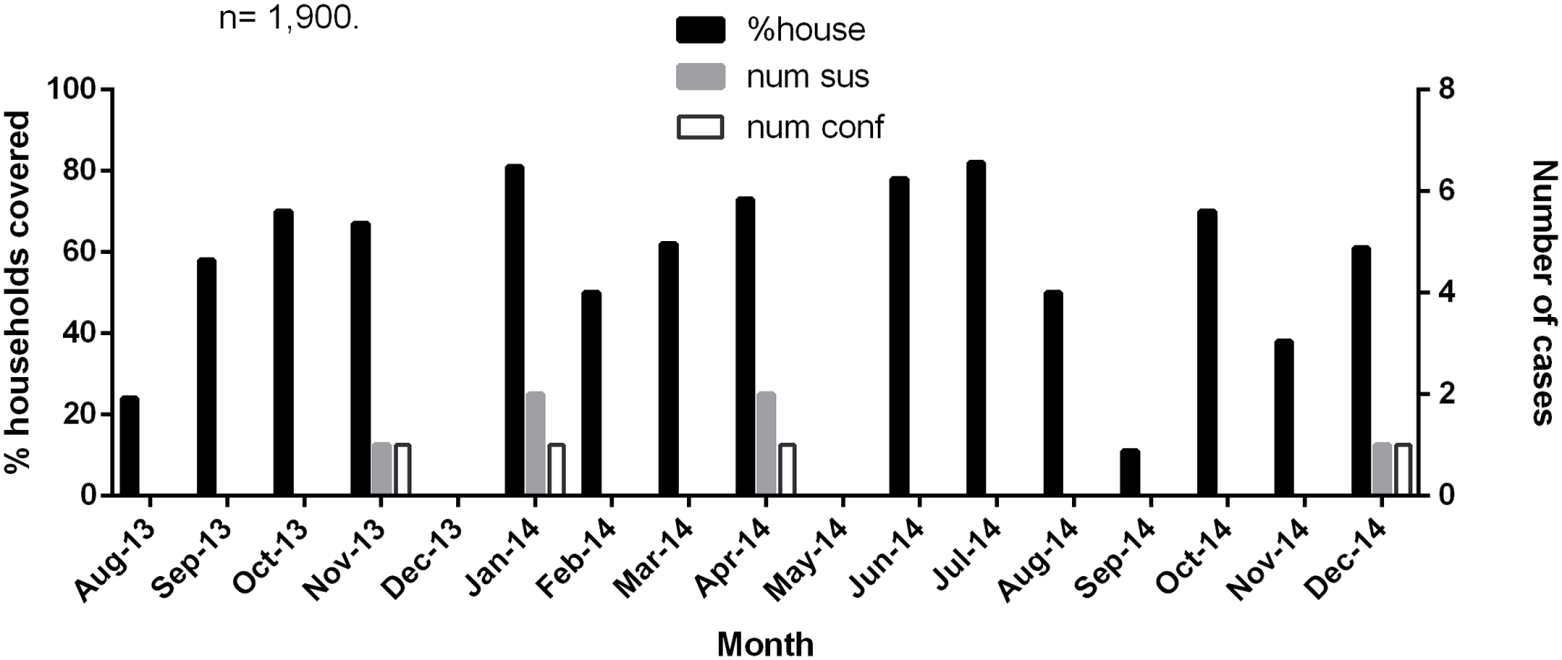
Monthly active surveillance of BU in the Achiase community. Graph shows data obtained from Achiase by monthly household visits. Black bars represent percentage of households covered by the volunteer, grey bars represent the number of suspected cases and white bars represent the number of laboratory confirmed cases.

The monthly household visits resulted in the detection of seven out of the eight laboratory confirmed cases. All seven cases were detected in three communities: Achiase (four cases), Ntobroso (two cases) and Akomfore (one case). Laboratory case confirmation rate for Achiase (4 out of 6 (66.7%)) was significantly higher (P = 0.03) than the combined rate recorded for all other communities (4 out of 23 (17.4%)). As shown in Fig 7, all four cases from Achiase were detected in months when the volunteer had covered more than 50% of the households.

## Discussion

Limited access of the population to health facilities and the reluctance of BU patients to seek medical care have made house-to-house surveys an attractive tool in studies on the disease epidemiology [13–15]. Here, we investigated recent anecdotal accounts of unstable *M*. *ulcerans* transmission along the Offin river.

We observed during our survey, scanty passive surveillance data which made our annual BU case trend analysis for each community a challenge. BU patients’ records preserved over time could also serve as reliable source of information for retrospective analyses which could add to the existing clinical knowledge of the disease. The importance of maintaining good patient records was demonstrated in a recent analysis of 1,227 BU case data collected from 2005 to 2011 in Benin. The study revealed a higher risk of developing osteomyelitis among male patients than female patients and a significant association between clinical presentation and development of permanent functional sequelae [16].

Inhabitants having both healed and active lesions were recorded in the study in order to account for historical trends as well as to get an overview of the current BU situation for each community. While only laboratory confirmed BU patients with active lesions were included, case definition for patients with healed lesions was based on clinical judgment. In view of the lack of passive surveillance data on BU for the study communities, findings of this study may thus serve as a reference for future longitudinal follow-up of the community.

In line with the decline in BU incidence in West Africa, Ghana recorded in 2014 nearly 50% less cases than in 2009 [2]. Along these lines, one key observation of this study is the overall decline in the prevalence of BU along the Offin river, even in known historically endemic communities. The high focal BU incidence recorded for Wromanso (K) in 2011 was perhaps due to an increase in community sensitization and awareness campaigns about BU conducted within the year. Consequently, this may have led to an increase in cases reporting to the health facilities or detection of more cases by the health staff through the awareness programs. However, some studies have reported similar upsurges in BU incidence in association with climatic and environmental changes [17–19].

In endemic communities of Africa, children have been reported as forming the majority of BU cases [20–25]. While children below 15 years of age formed nearly half of all cases detected in our study, we observed an underrepresentation of children below 5 years among cases, consistent with the findings of a recent study conducted in Cameroon [14]. Moreover, sero-epidemiological studies in Ghana and Cameroon demonstrated that children of this age group were less exposed to *M*. *ulcerans* [26].

Consistent with findings of a previous study conducted along the Densu river in Ghana [13], we observed a very low prevalence of BU upstream the river whereas mid and downstream communities recorded high prevalence. Similar to the repeated association of BU with man-made modification of aquatic environments [11,17,18,23,27–29], the intense gold mining activities observed in our study area were also localized mid and downstream the river. Recently, a detailed study on land cover and its association with BU in the downstream region of the Offin demonstrated a significant association between mining and the occurrence of the disease [10]. In the same study, the distribution of alluvial gold mining patches in areas of BU foci was made evident using high resolution satellite images. The low-lying feature of the mid and downstream regions of the river also supports previously reported association of BU with landscapes of low elevation [30,31,28].

The monthly household-based surveillance conducted by the community volunteers in the course of this study resulted in the detection of seven of the eight laboratory confirmed active BU cases, all of whom had early stage lesions. This underscores the important role of community volunteers in early case detection and uncomplicated treatment of BU [32–34]. However, ideally, only category I lesions should have been detected considering that active surveillance strategies were employed to monitor the emergence of new cases after the exhaustive case search. This could be explained that some early lesions were missed since the volunteers couldn’t consistently achieve 100% household coverage. Alternatively some patients may have ignored the very early clinical BU signs and may have only presented themselves to the volunteers on such visits when lesions have progressed to category II. As reviewed [35] and previously reported [36] patients with large active lesions may play a role in transmission of *M*. *ulcerans* by disseminating the bacteria from their active lesions into the environment which may then serve as a source of infection to others. This implies that intense continuous early case detection and timely antibiotic treatment in an endemic area may result in the interruption of this cycle leading to the gradual decline of cases, as observed for example in Ghana and Benin.

The current active surveillance approach employed by the BU control programs is mainly community based. One cost effective way to sustain the monthly household surveillance will be to integrate the BU surveillance with other prevalent skin diseases like yaws, cutaneous leishmaniasis and leprosy [37]. As a tool, the mobile phone data collection can serve as a back-up to address the gaps in data collected at the health facility or district levels.

## Supporting Information

S1 Checklist STROBE Checklist. (DOC)
